# Indication of ongoing amphipod speciation in Lake Baikal by genetic structures within endemic species

**DOI:** 10.1186/s12862-019-1470-8

**Published:** 2019-07-08

**Authors:** Anton Gurkov, Lorena Rivarola-Duarte, Daria Bedulina, Irene Fernández Casas, Hendrik Michael, Polina Drozdova, Anna Nazarova, Ekaterina Govorukhina, Maxim Timofeyev, Peter F. Stadler, Till Luckenbach

**Affiliations:** 1 Irkutsk State University, Karl Marx st. 1, 664003 Irkutsk, Russia; 2 Baikal Research Centre, Lenin st. 21, Irkutsk, 664003 Russia; 3 Interdisciplinary Center for Bioinformatics, University of Leipzig, Härtelstraße 16-18, D-04107 Leipzig, Germany; 4 Department of Bioanalytical Ecotoxicology, UFZ – Helmholtz Centre for Environmental Research, Permoserstraße 15, D-04318 Leipzig, Germany

**Keywords:** Amphipods, Baikal, COI, Cryptic species, Speciation

## Abstract

**Background:**

The ancient Lake Baikal is characterized by an outstanding diversity of endemic faunas with more than 350 amphipod species and subspecies. We determined the genetic diversity within the endemic littoral amphipod species *Eulimnogammarus verrucosus*, *E. cyaneus* and *E. vittatus* and investigated whether within those species genetically separate populations occur across Lake Baikal. *Gammarus lacustris* from water bodies in the Baikal area was examined for comparison.

**Results:**

Genetic diversities within a species were determined based on fragments of cytochrome c oxidase I (COI) and for *E. verrucosus* additionally of 18S rDNA. Highly location-specific haplogroups of *E. verrucosus* and *E. vittatus* were found at the southern and western shores of Baikal that are separated by the Angara River outflow; *E. verrucosus* from the eastern shore formed a further, clearly distinct haplotype cluster possibly confined by the Selenga River and Angarskiy Sor deltas. The genetic diversities within these haplogroups were lower than between the different haplogroups. Intraspecific genetic diversities within *E. verrucosus* and *E. vittatus* with 13 and 10%, respectively, were similar to interspecies differences indicating the occurrence of cryptic, morphologically highly similar species; for *E. verrucosus* this was confirmed with 18S rDNA. The haplotypes of *E. cyaneus* and *G. lacustris* specimens were with intraspecific genetic distances of 3 and 2%, respectively, more homogeneous indicating no or only recent disruption of gene flow of *E. cyaneus* across Baikal and recent colonization of water bodies around Baikal by *G. lacustris*.

**Conclusions:**

Our finding of separation of subgroups of Baikal endemic amphipods to different degrees points to a species-specific ability of dispersal across areas with adverse conditions and to potential geographical dispersal barriers in Lake Baikal.

**Electronic supplementary material:**

The online version of this article (10.1186/s12862-019-1470-8) contains supplementary material, which is available to authorized users.

## Background

**Fig. 1:**
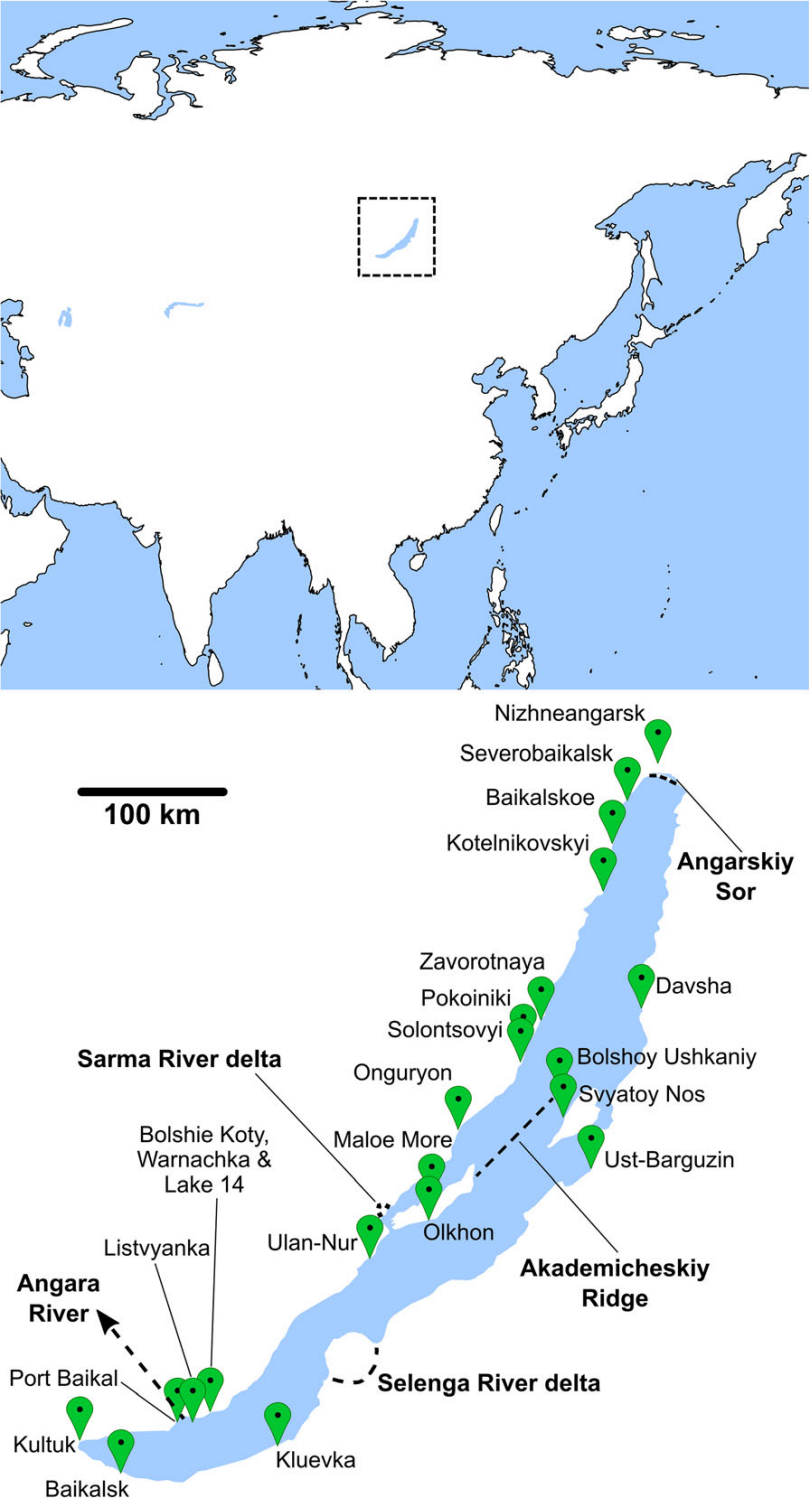
Maps showing the geographical position of Lake Baikal in Asia (above) and the sampling points of the four studied amphipod species at Lake Baikal (below). Depicted are also major river inflows (Selenga River delta; Angarskiy Sor that comprises the inflows of the Upper Angara and Kichera Rivers; Sarma River delta) and the Angara River outflow. The Akademicheskiy Ridge is an underwater shelf separating basins in northern and central Baikal. See Additional file 4: Figure S1 for the detailed bathymetric chart of Lake Baikal. Maps based on freely available cartographic material from Natural Earth (see Materials and Methods)

Lake Baikal in Eastern Siberia (Fig. 1; Additional file 4: Figure S1) is by volume (23,000 km^3^) and depth (maximum depth: 1642 m) the largest freshwater lake in the world [1]. It is a hotspot of aquatic animal speciation and in Baikal approximately 60% of the ~ 2600 animal species are endemics [2]. The highly diverse endemic animal communities are confined to Baikal and are distinct from those of water systems in Baikal’s vicinity that are inhabited by a fauna common in the Holarctic [3]. The long geological history of the lake, which with 25–30 million years is one of the most ancient freshwater lakes in the world, may be a critical precondition for the evolution of Baikal’s exceptional biodiversity [1].

**Fig. 2:**
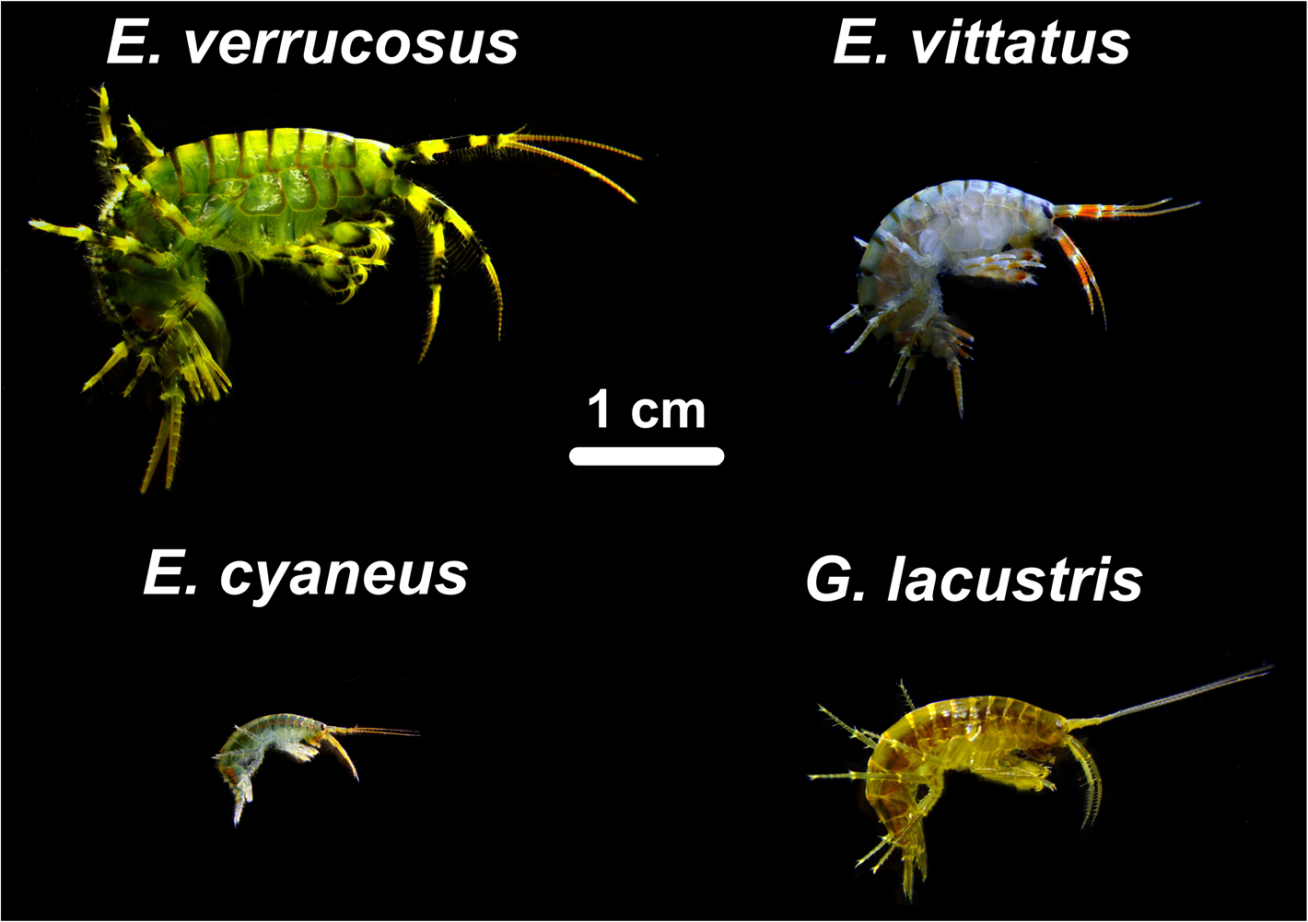
Photographs of adults of the studied amphipod species: Lake Baikal endemics *Eulimnogammarus verrucosus*, *E. vittatus* and *E. cyaneus* and the Holarctic species *Gammarus lacustris*

The amphipods (Amphipoda, Crustacea) of Baikal are particularly species-rich, with so far 354 species and subspecies described. They show a considerable morphological and ecological diversity and inhabit benthic substrates in all water depths of Baikal and its only outflow, the Angara River [4]. The high amphipod biodiversity of Baikal can partially be explained with a high rate of evolution that potentially is due to an elevated mutation rate [5–7]. Baikal amphipods offer a unique opportunity for following ongoing microevolution resulting in phenotypically clearly distinguishable species (Fig. 2).

The formation of separate populations among which gene flow is limited can promote the formation of new species. For littoral amphipod species of Baikal the existence of geographically distinct populations was indicated in studies based on allozyme analysis of *Eulimnogammarus cyaneus* [8] and of DNA sequence comparisons of a COI (cytochrome c oxidase I) gene fragment of *Gmelinoides fasciatus* (Stebbing, 1899) [9, 10].

We here aimed to reveal the intra- and inter-species genetic diversity of endemic amphipods by genetically comparing multiple specimens from each species per site from a wide range of sampling locations across the lake. Overall, four amphipod species were investigated, three from the endemic Baikal genus *Eulimnogammarus*, *E. cyaneus*, *E. verrucosus* and *E. vittatus*, and the Holarctic amphipod *Gammarus lacustris* (Fig. 2). The *Eulimnogammarus* species inhabit the littoral zone of Lake Baikal, are common across the lake, are easily accessible for sampling and have distinct morphological features enabling unambiguous species determination. *Gammarus lacustris* has a similar habitat as the *Eulimnogammarus* species but it does not occur at the open shores of Lake Baikal but in sheltered shallow bays of Baikal and in water bodies in the close vicinity of the lake [4, 11]. The genetic diversity of *G. lacustris* specimens from separate water bodies around Baikal was investigated for comparison to the Baikal species. The exchange of *G. lacustris* individuals *via* active migration between those water bodies can be ruled out because the water bodies are not connected. The genetic diversity was explored based on DNA sequence alignments of a COI gene fragment. Previous population structure analyses of littoral and deep-water amphipod species from Baikal based on COI gene sequence alignments [5, 9] served as reference for our analyses. Additionally, 18S rDNA sequences from *E. verrucosus* specimens were compared to corroborate findings obtained with COI sequences of this species.

## Results

### Interspecific COI sequence variability

The genetic distances (uncorrected Hamming dissimilarity) of *E. verrucosus* to the other species are: *E. vittatus*: 13–16% < *E. cyaneus*: 15–19% < *G. lacustris*: 20–23%; genetic distances to *E. cyaneus* are: *E. vittatus*: 16–18% < *G. lacustris*: 20–23%; and between *E. vittatus* and *G. lacustris*: 21–22%.

The numbers of investigated specimens were: *E. verrucosus* – 216 from 20 sampling sites, *E. cyaneus* – 155 from 16 sampling sites, *E. vittatus* – 22 from three sampling sites, *G. lacustris* – 32 from three sampling sites (refer also to Table 1 and Additional file 1: Table S1).

**Table 1:** Numbers of sampled amphipod specimens at the different sampling sites at Lake Baikal and at waters in the close vicinity of Baikal (for *G. lacustris*). Refer to the map in Fig. 1 for the geographical locations of the sites

Sampling sites	*Eulimnogammarus verrucosus*	*Eulimnogammarus vittatus*	*Eulimnogammarus cyaneus*	*Gammarus lacustris*
Baikalsk	11	7	8	
Baikalskoe	11		10	
Bolshie Koty	31	9	19	
Bolshoy Ushkaniy	11		10	
Davsha	6			
Kluevka			8	
Kotelnikovskyi	10		3	
Kultuk	12			
Listvyanka	11	6	6	
Maloe More	21		19	
Olkhon	8			
Onguryon, open Baikal	10		11	
Pokoiniki	6		10	
Port Baikal	10		11	
Severobaikalsk	13		10	
Solontsovyi	11		2	
Svyatoy Nos	11		10	
Ulan-Nur	3		8	
Ust-Barguzin	6			
Warnachka	9			
Zavorotnaya	5		10	
Lake 14				11
Nizhneangarsk				11
Onguryon, pond				10
*Total number of sampled amphipods*	216	22	155	32

### Intraspecific COI sequence variability

#### Haplotype patterns – entire Lake Baikal

**Fig. 3:**
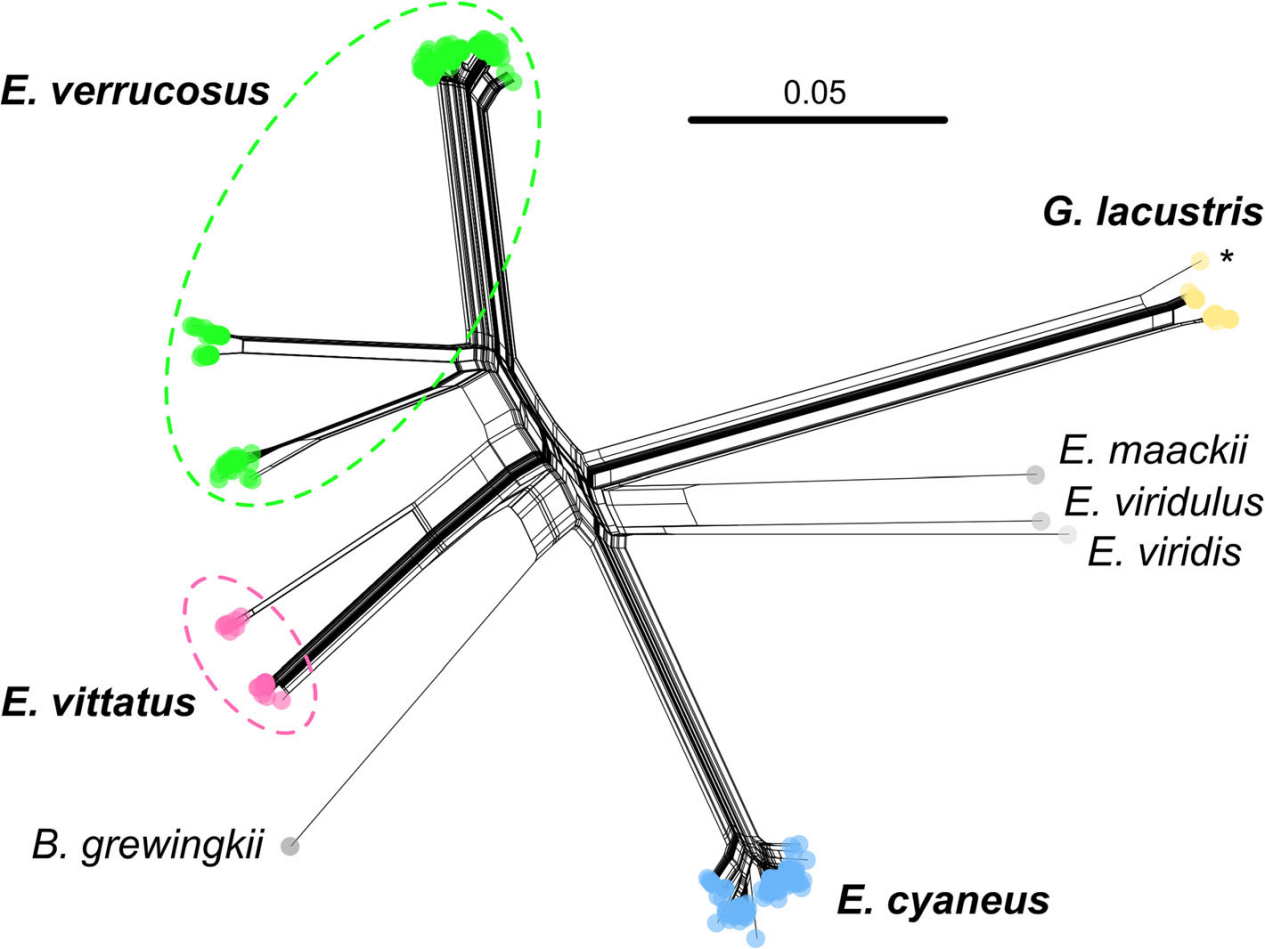
Phylogenetic network based on the alignment of corresponding COI sequence fragments from the four studied species *Eulimnogammarus verrucosus*, *E. vittatus*, *E. cyaneus* and *Gammarus lacustris*. Respective COI sequences from *E. maackii* (AY926663.1), *E. viridulus* (AY926665.1), *E. viridis* (AY926664.1) [16] and *Brachyuropus grewingkii* (NC_026309.1) [6] serve as outgroups. The scale bar indicates the number of substitutions per base pair. The asterisk designates a sample of *G. lacustris* (GU066811.1) from Yellowstone Lake, USA [61]. See Additional file 5: Fig. S2 for the maximum likelihood tree of these sequences

The phylogenetic network constructed on the basis of the COI sequence fragments from specimens from all four amphipod species (for sequence data refer to the Additional file 2) shows considerable intraspecific differences of haplotypes of *E. verrucosus* and *E. vittatus* specimens with clearly separate haplogroups; in contrast, the COI sequences of *E. cyaneus* and *G. lacustris* specimens from across the lake were overall more similar and for each species appeared in one single branch in the phylogenetic network (Fig. 3, Additional file 5: Figure S2). The maximum intraspecific genetic distances within *E. verrucosus* and *E. vittatus* reached 13 and 10%, respectively, which is similar to the interspecific variation between these two species (Fig. 3). Genetic distances of specimens within the separate clusters for those two species were 2–4%. The genetic distances of specimens within *E. cyaneus* and *G. lacustris* did not exceed 3 and 2%, respectively. For *G. fasciatus*, another littoral amphipod from Baikal, we calculated an intraspecific variability of 8% from data on the COI fragment obtained earlier [9].

**Fig. 4:**
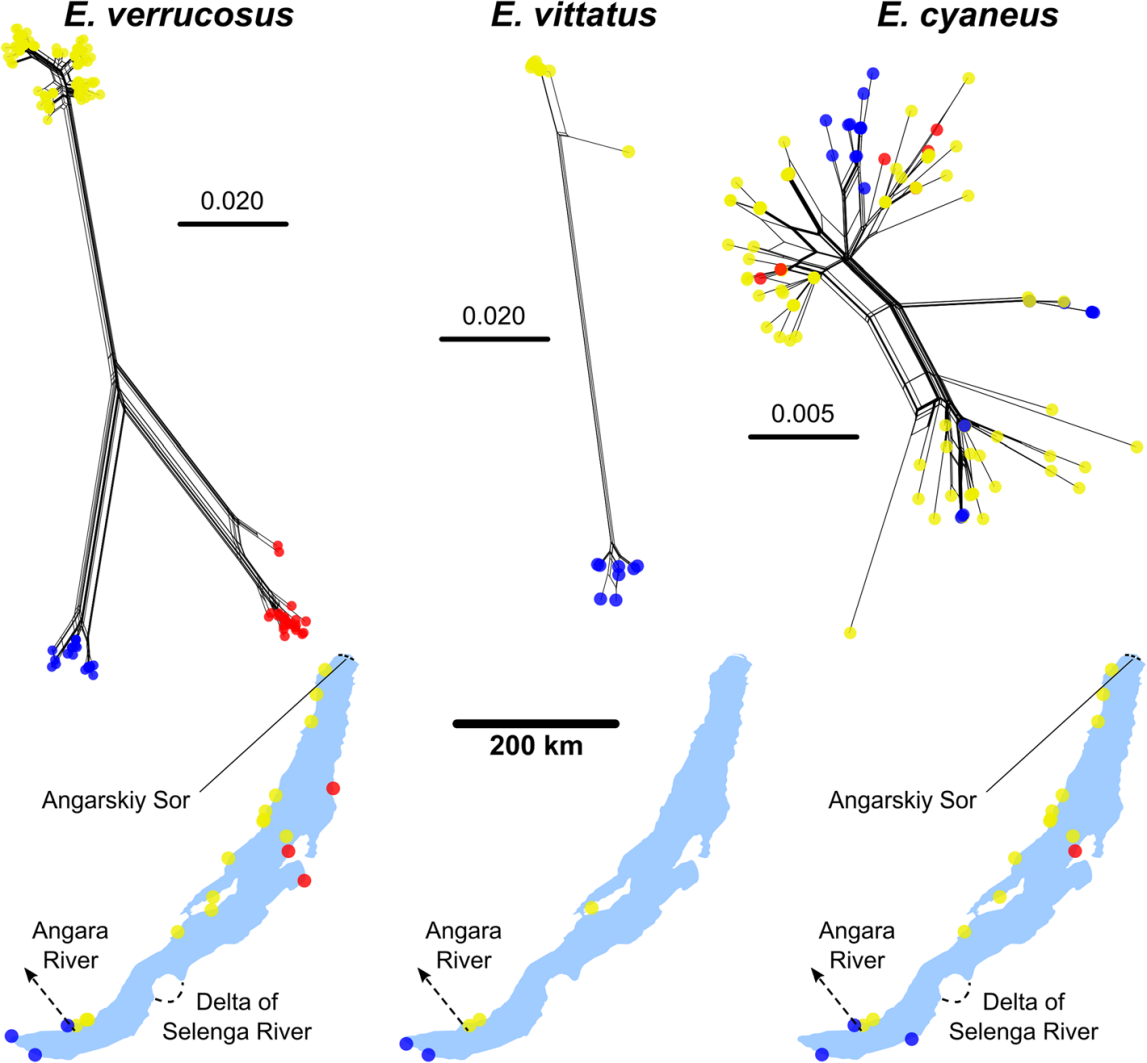
Phylogenetic networks based on DNA sequence comparisons of a COI fragment from amphipods endemic to Lake Baikal. Sampling locations of specimens from the different clusters are indicated on the map as yellow, blue and red circles. The scale bars indicate the numbers of substitutions per base pair. Note different scales. The network for the *E. vittatus* COI fragments includes sequences AY926666.1 (separated branch in the upper cluster of the network) [16] sampled close to Maloe More (western region) and NC_025564.1 [62] sampled close to Kultuk (southern region). Maps based on freely available cartographic material from Natural Earth (see Materials and Methods)

The separate haplotype clusters of *E. verrucosus* and *E. vittatus* specimens revealed by the phylogenetic networks based on the COI fragments coincide with different regions of sampling locations (Fig. 4). In contrast, the haplotypes of *E. cyaneus* specimens did not depend on geographical provenance of specimens (Fig. 4).

The three major *E. verrucosus* haplotype clusters can be allocated to specimens sampled at the shores in the western and north-western parts; the eastern and northeastern parts; and to the southern parts of Baikal. The outflow of the Angara River in the southwest separates the western and southern regions. Two other major geographical structures potentially functioning as dispersal barriers are the delta of the Upper Angara and Kichera Rivers (Angarskiy Sor) in the north separating the northwestern and northeastern regions and the delta of the Selenga River in the southeast, the main water inflow of Lake Baikal, separating the eastern and southern regions.

**Fig. 5:**
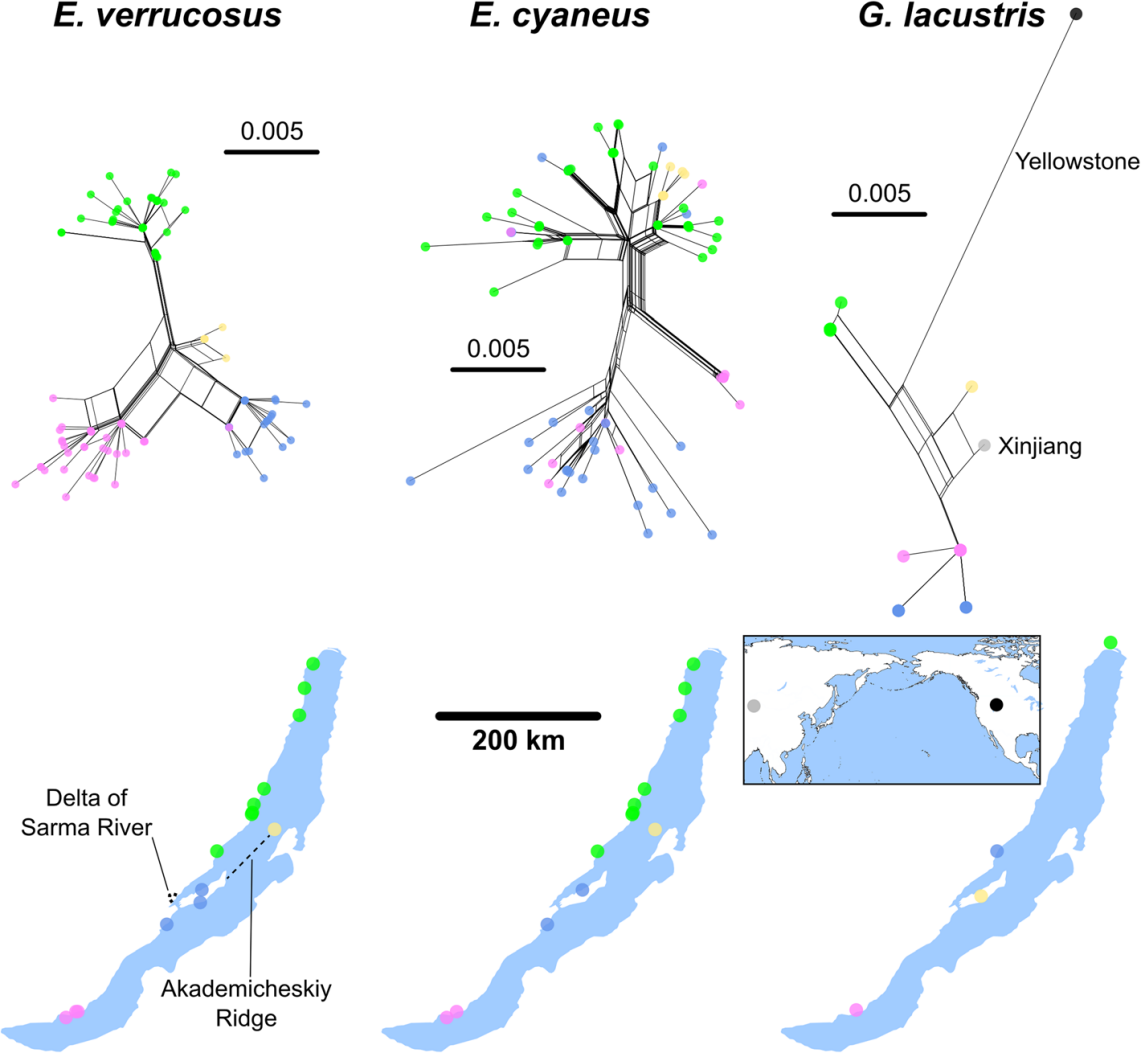
Phylogenetic networks based on DNA sequence comparisons of a COI fragment from *E. verrucosus*, *E. cyaneus* and *G. lacustris* specimens from sampling points at the western shore of Lake Baikal from the entire stretch from Listvyanka bay (northern shore of the Angara River outflow) up to Severobaikalsk and at Olkhon and Bolshoy Ushkaniy islands. The scale bars indicate the numbers of substitutions per base pair (the scale is identical for all species). The network with *G. lacustris* sequences also contains COI fragments from specimens from a pond on Olkhon Island (AY926671.1) [16]; from Xinjiang, China (JF965916.1) [63] and from Yellowstone Lake, USA (GU066811.1) [61]. Maps based on freely available cartographic material from Natural Earth (see Materials and Methods)

Three clusters of COI haplotypes can be distinguished within the phylogenetic network for *E. cyaneus* (Figs. 4 and 5). Sequences from specimens from the southern region (sampling points between Port Baikal to the Kluevka settlement) and the southern part of the western region (bays at Listvyanka at the northern riverside of the Angara River and at Bolshie Koty) appear in all three clusters; sequences from specimens from the central part of the western region (sampling points: Ulan-Nur Cape and Maloe More Strait) appear in the two large clusters; and sequences from specimens from the entire northern half of Lake Baikal (sampling points from Onguryon settlement to Severobaikalsk town in the western region, on the Svyatoy Nos Peninsula on the eastern shore and on Bolshoy Ushkaniy Island) appear only in the upper large cluster (Fig. 4). Accordingly, the maximum genetic diversity of specimens from the northern region was with 2% slightly lower than for all *E. cyaneus* specimens from the entire lake. Analysis of the COI sequence alignments using SAMOVA did not indicate apparent grouping of *E. cyaneus* sampling sites across the entire lake: *F*_CT_ showed no global maximum, it gradually increased with increasing *K* (up to 15 groups). However, a local *F*_CT_ maximum occurred at *K* = 3 (*F*_CT_ = 0.3552; *F*_CT_ = 0.3256 with *K* = 2, *F*_CT_ = 0.3549 with *K* = 4) both with and without constraints for the geographic relations of the locations. With *K* = 3, SAMOVA indicated the following groups: one associated with the locations in the north region (Svyatoy Nos, Bolshoy Ushkaniy and from Onguryon to Severobaikalsk), one at the sampling points Baikalsk and Kluevka in the south, and one associated with the sampling points from Port Baikal to Maloe More at the west coast.

#### Haplotype patterns – western Lake Baikal region

Since specimens from the western region (sampling points: from Listvyanka bay to Severobaikalsk town; approximate distance: ~ 500 km) were available from a comparatively high number of sampling points and the *G. lacustris* sampling points were spatially close to the most northern and most southern *Eulimnogammarus* sampling points of the western region data for the respective specimens were more closely examined and compared with the *G. lacustris* haplotype data (Fig. 5).

The phylogenetic network with *E. cyaneus* haplotypes from this region of Lake Baikal (Fig. 5) shows a structure and haplotype distribution that is equal to that when including the haplotypes from the entire lake in the analysis (Fig. 4); the genetic distances among haplotypes from the western region and from the entire lake are correspondingly 3%. For *E. verrucosus*, in contrast, the genetic distances between haplotypes from the western region equal only 2% which is considerably less than the maximally 13% genetic distances when comparing the haplotypes across the entire lake.

The haplotypes of *E. verrucosus* and *E. cyaneus* specimens sampled in different years at Bolshie Koty bay and Maloe More Strait were located at equal positions in the phylogenetic networks indicating little migration activities of individuals of the different haplotypes across the lake over the considered time.

The *E. verrucosus* haplotypes from the western region show the following structure (Fig. 5): The haplotypes of individuals from the sampling points of the southern part of the western shore (Listvyanka Bay, Warnachka Bay and Bolshie Koty Bay) form a tight cluster with no regional subclusters on the network. Haplotypes of *E. verrucosus* specimens from Ulan-Nur Cape (central Baikal) and both sampling points on Olkhon Island form a tight cluster which is close to a haplotype cluster of specimens from Bolshoy Ushkaniy Island; however, haplotypes of four individuals from Olkhon Island are identical to a haplotype sampled at Bolshie Koty Bay. The haplotypes of specimens sampled between the Onguryon settlement to Severobaikalsk (the northern part of the western shore, north of the Sarma River delta) form a subcluster slightly separated from the other western region haplotypes. SAMOVA performed for these sampling locations confirmed the observed pattern (both with and without constraint for the geographic relations of the locations). The *F*_CT_ value reached a plateau (*F*_CT_ = 0.776) at *K* = 4 clusters of sampling locations (*F*_CT_ = 0.710 at *K* = 3, *F*_CT_ = 0.777 at *K* = 5) although the absolute maximum value was found for *K* = 6 (*F*_CT_ = 0.781). With *K* = 4, the grouping along the west coast was the following: Listvyanka, Warnachka, Bolshie Koty; Ulan-Nur, Maloe More, Olkhon; Bolshoy Ushkaniy; from Onguryon to Severobaikalsk. With *K* = 6, Olkhon and Onguryon formed separate clusters.

In the comparison of *G. lacustris* COI sequence fragments samples from three water bodies along the western shore of Lake Baikal were included together with previously obtained samples from a pond on Olkhon Island and from a geographically distant water body in Xinjiang (China) and from the Yellowstone Lake (USA) (Fig. 5). The COI sequence from Xinjiang falls within the cluster of the specimens from the Baikal Region that show maximum genetic distances of 2%. The sequence from the specimen from Yellowstone Lake is more separate from the other sequences; with this sequence included the genetic variability within *G. lacustris* COI sequences was up to 4%. In contrast to the Baikal amphipods, geographical patterns regarding distribution and relation of *G. lacustris* haplotypes were not seen (Fig. 5).

### Comparative metrics for the COI alignments

The comparison of dN/dS values demonstrated similar levels of stabilizing selection for all analyzed species: 0.02 for *E. verrucosus*, approximately 0.04 for both *E. vittatus* and *E. cyaneus* and 0.06 for *G. lacustris*. For the littoral Baikalian amphipod *Gmelinoides fasciatus* we determined a dN/dS value of 0.12 using data from [9]. Thus, the variability in intraspecific genetic distances among different amphipod species of the Baikal littoral zone cannot be related to any kind of positive selection on the level of the translated protein.

For assessing relative effective population sizes, the effective number of codons (ENC) was determined. For the western, southern and eastern sample groups of *E. cyaneus*, ENC values ranged between 42.9–47.6 with an overall median of 44.6 and *p*-values were > 0.05 indicating no separation of populations across Baikal. For *E. verrucosus*, the eastern sample group formed a population significantly different from the western and southern sample groups (both *p*-values < 10^− 8^) with ENC values ranging from 44.8–48.8 with a median of 48.0. For the western and southern *E. verrucosus* sample groups, ENC values with an overall range of 41.6–47.3 with a median of 44.1 were not significantly different (*p*-value = 0.49) and were similar to ENC values of the western and southern *E. cyaneus* sample groups (both *p*-values = 0.63). Comparing the eastern population of *E. verrucosus* with *E. cyaneus* inhabiting the same part of the lake, ENC values were also significantly different (*p*-value < 10^− 4^) but this comparison may be biased due to lower sampling coverage for *E. cyaneus*. This result indicates that *E. verrucosus* and *E. cyaneus* have overall similar effective population sizes; the effective size of the eastern *E. verrucosus* population, in contrast, may be smaller than for *E. cyaneus* and for other populations of *E. verrucosus* [12].

Analysis of the COI sequence data with ABGD indicated that the separate *E. verrucosus* and *E. vittatus* haplotype clusters are on a species level [13]; a separation of subgroups into species was not seen within *E. cyaneus*. For *G. lacustris*, a separation of two groups was indicated by ABGD; this is likely an artifact as the number of sampling sites was low.

### COI mutation rates within the *Eulimnogammarus* species

Mutation rates within the COI fragment of *E. cyaneus* were compared with those of *E. verrucosus* and *E. vittatus*. In comparison to *E. cyaneus*, the mutation rate was higher in *E. verrucosus* as indicated by the relaxed clock coalescent model (1.443 *vs.* 1.005) and lower in *E. vittatus* (0.934 *vs.* 1.225). However, in both pairs the model comparison demonstrated no need for rejecting the null hypothesis of equal substitution rates: evidence for relaxed clock model in the pair *E. verrucosus* / *E. cyaneus* was anecdotal (path sampling BF_RS_ = 1.6, stepping-stone sampling BF_RS_ = 1.5) according to the system of [14] and in the pair *E. vittatus* / *E. cyaneus* the strict clock model has from moderate to strong evidence (path sampling BF_RS_ = 0.10, stepping-stone sampling BF_RS_ = 0.09). Thus, it can be assumed that the three studied *Eulimnogammarus* species have identical mutation rates.

### Intraspecific 18S rDNA sequence variability of *E. verrucosus*

**Fig. 6:**
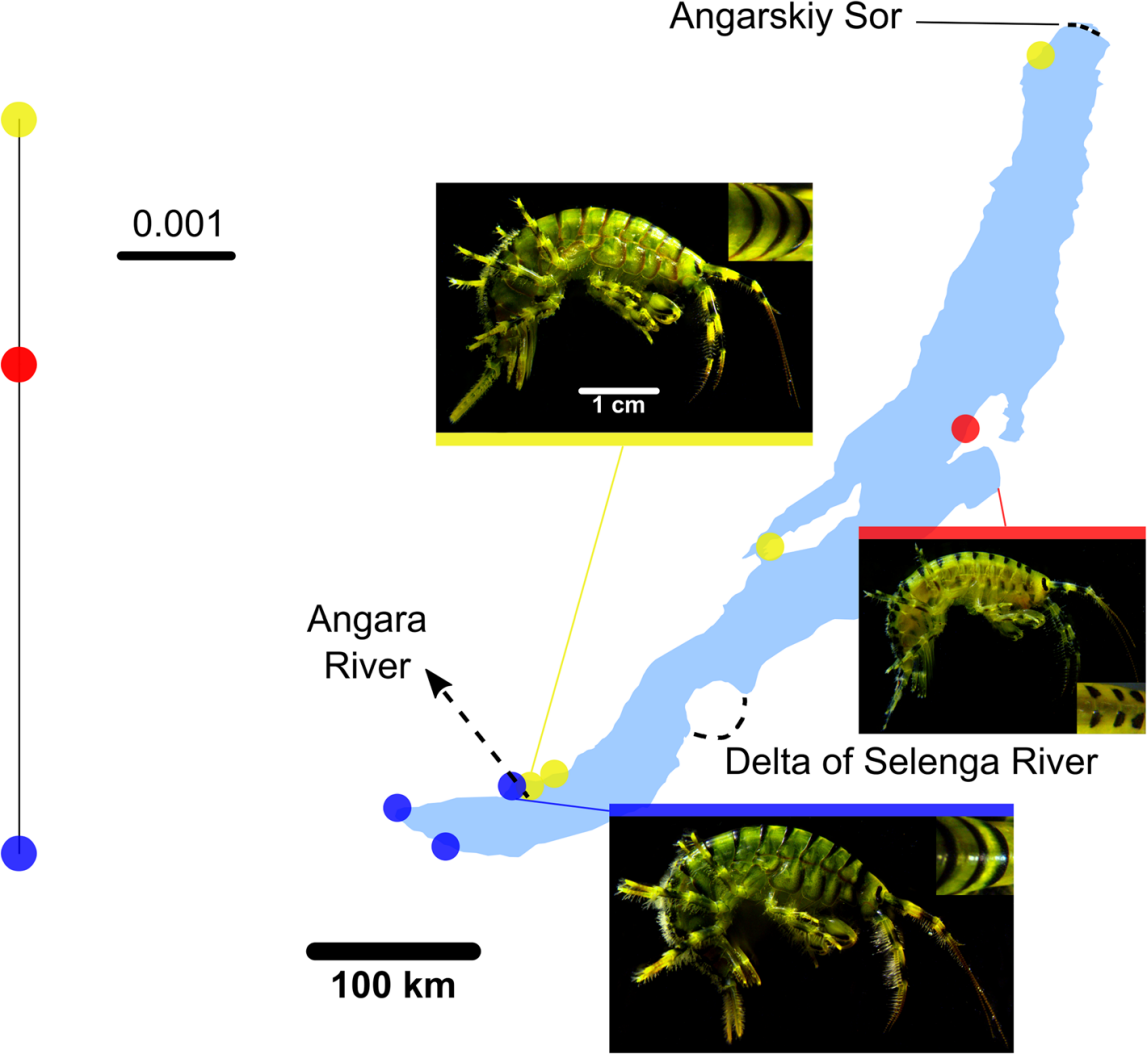
Phylogenetic network (left) based on a 18S rDNA sequence fragment from *E. verrucosus* specimens from sampling points in the southern, western and eastern regions of Baikal and photographs of *E. verrucosus* specimens from each haplotype from respective sampling points (right). Note the differences in the shape of the stripes on the closeup images of the dorsal crest (the first three segments are displayed). The scale on the photographs of the entire specimens is identical; the sizes of the photographed individuals are not representative for the individuals of the respective haplotypes. The scale bar of the network indicates the number of substitutions per base pair. The network comprises the data from seven samples from the western (including sequence AY926773.1) [16], five samples from the southern and two samples from the eastern regions of Lake Baikal. See Additional file 6: Figure S3 for the maximum likelihood tree of these sequences. Maps based on freely available cartographic material from Natural Earth (see Materials and Methods)

To determine to which degree the three distinct haplogroups that were seen based on COI sequence comparisons in *E. verrucosus* can be confirmed by another, nuclear genetic marker, 18S rDNA sequences from *E. verrucosus* individuals from the three sampling points Listvyanka, Port Baikal and Svyatoy Nos were also compared (for sequence data refer to Additional file 3). The separation of genetically clearly distinct western, eastern and southern *E. verrucosus* groups across Baikal was confirmed by 18S rDNA sequence comparisons (Fig. 6, Additional file 6: Figure S3). Although 18S rDNA sequence analysis was performed only for a few specimens per location this pattern was also seen. Within sample groups the 18S rDNA sequence fragments were identical but across sample groups sequences differed in two to four positions.

### Morphological features of *E. verrucosus* specimens from different locations

Morphologies of *E. verrucosus* specimens from the different genetic clusters were examined for determining to which extent they morphologically differ. Specimens of *E. verrucosus* stored in ethanol since sampling in 2012 from the following sampling locations were morphologically closely examined: Listvyanka, Bolshie Koty, Maloe More, Severobaikalsk, Bolshoy Ushkaniy (western haplogroup), Port Baikal, Baikalsk (southern haplogroup) and Svyatoy Nos (eastern haplogroup). Per sampling site five specimens were available in this sample set and additional individuals were collected in 2018 to increase the sample size for each haplotype at the sites Listvyanka (*n* > 50), Port Baikal (n > 50) and Ust-Barguzin (*n* = 20) (Fig. 6).

The species identity of all specimens as *E. verrucosus* was confirmed as they showed the following distinctive features according to [15]: (a) All segments of the metasome and urosome are dorsally covered with spines on comb-shaped elevations and with numerous setae. (b) The antennae 1 are shorter than the body and longer than the antennae 2; the peduncles of antennae 1 are shorter than the ones of the antennae 2. (c) The main segment of the lower antennae has individual setae and a group of short spines on the lower frontal corner. (d) Subchelae of the second pair of gnathopods are shorter than ones of the first pair. (e) The basipodites of pereopod 5 are almost rectangular; the basipodites of pereopods 6 and 7 are narrow and pear-shaped, and their posterior margins are concave and have no or a rudimentary lobe at the distal part (this feature is absent in some specimens from Severobaikalsk, Svyatoy Nos and Ust-Barguzin). (f) The uropods 3 have a long, single-segment outer branch covered with short spines and simple setae (instead of simple setae some specimens from the southern and eastern sample groups from sampling points Bolshoy Ushkaniy, Port Baikal, Baikalsk and Svyatoy Nos have pinnate setae); the inner branch is ten times shorter than the outer branch. (g) The telson is separated up to the basis; the branches are heart-shaped and covered by spines and setae at the terminal part.

However, specimens from the eastern sample group show two clearly distinct morphological features not found in specimens from the other two haplotype clusters: (1) The amounts of setae on the dorsal parts of the metasome and the urosome are evidently lower in specimens from both Svyatoy Nos and Ust-Barguzin; (2) the black stripes along the caudal edges of the body segments of the individuals from the eastern genetic group were intermittent at the dorsal crest but continuous in the specimens from the other two haplogroups (Fig. 6). This feature could only be seen in individuals from Ust-Barguzin but not in specimens from Svyatoy Nos due to bleaching during storage.

## Discussion

In this study the question was addressed whether genetically separate populations of endemic littoral amphipods from the *Eulimnogammarus* genus from across Lake Baikal can be distinguished. The examined species are abundant and their habitat is common in the littoral zone of Baikal. The aim of this study was to determine to which degree and in which regions dispersal of individuals and gene flow among amphipod populations takes place and to identify potential geographical barriers. At some sites samplings were performed in subsequent years showing that the haplotype pattern at the respective sampling points was stable over multiple samplings indicating no rapid gene flow due to migration of amphipods between haplogroup regions (refer to above section “Intraspecific COI sequence variability”).

Phylogenetic networks of the sequences indicated three clearly different haplogroups specific for a western, eastern and southern population for the species *E. verrucosus* and two different haplogroups specific for western and southern populations for *E. vittatus*. Genetic distances between the different haplogroups were as large as between different amphipod species (Fig. 3). The western haplogroup of *E. verrucosus* showed further separation into at least four subclusters. In contrast, the genetic structure across the sampled *E. cyaneus* individuals was in comparison highly homogenous. Maximum genetic distances among *E. verrucosus* and *E. vittatus* individuals exceeded that within *E. cyaneus* by more than four and three times, respectively (Figs. 4, 5); genetic distances between *G. lacustris* individuals were similar to those seen for *E. cyaneus*. The association of the *E. vittatus* and *E. verrucosus* haplotype groups to separate populations as indicated by the phylogenetic networks was further shown by ABGD analysis. The obtained COI phylogenetic network (Fig. 3; Additional file 5: Figure S2) concurs with the previously described phylogenetic relations of the studied species [7, 16]. A clear separation of populations from different regions based on the COI gene fragment was previously also shown for *Gmelinoides fasciatus*, another littoral amphipod from Lake Baikal with a similar geographical coverage as the here studied *Eulimnogammarus* species [9]. The intraspecific variability of the COI fragment of up to 8% was 40 and 20% lower than that found here for *E. verrucosus* and *E. vittatus*, respectively.

It was an interesting finding that *E. verrucosus* specimens sampled at the Bolshoy Ushkaniy Island belong to the western haplotype cluster although the island is geographically closer to the eastern shore. This indicates that geographical structures existed in the recent past providing conditions, such as sufficiently shallow water, e.g. at the Akademicheskiy Ridge that separates basins in northern and central Baikal (Fig. 5; Additional file 4: Figure S1) where the water was much shallower in the past, and/or the absence of strong currents, allowing accessibility of the island for *E. verrucosus* from the western shore. Access to the island from the eastern shore (i.e. Svyatoy Nos Peninsula), however, appears to be blocked for *E. verrucosus* by open Baikal (Additional file 4: Figure S1). Indeed, there are indications that the Akademicheskiy Ridge was above the water surface until the Late Pleistocene (approximately 100,000–150,000 years ago) [17]. Thus, until that time the ridge could have provided a littoral habitat zone for *E. verrucosus* along which gene flow between the western shores and Bolshoy Ushkaniy Island could have been maintained. The evolutionary rates of mtDNA in different animal taxa were indicated by studies e.g. on the *Drosophila obscura* group [18] and on humans and chimpanzees [19] examining COI sequence differences. Based on those studies it may be assumed that a separation of *E. verrucosus* from the west coast and from Bolshoy Ushkaniy Island for 150,000 years should result in a genetic divergence of < 1%, which would be in agreement with our observation.

A previous study indicated a separation of *E. cyaneus* populations inhabiting the northern and southern halves of Baikal based on allozyme analysis [8]. Our data partially support this observation by demonstrating that the COI haplotype diversity in *E. cyaneus* from the northern regions of Lake Baikal is reduced. This may also be indicated by the results from the SAMOVA analysis with *K* = 3; however, the separation of the group of sampling points Baikalsk and Kluevka does not agree with the allozyme data. The differences in the outcomes of the allozyme [8] and our study indicate that the COI sequences are more conserved than the allozymes examined in [8] and therefore less sensitive for indicating separation of populations of this species.

The genetic diversity of *G. lacustris* specimens from the Baikal region was considerably lower than that of the other studied species and there was no association of haplotypes with geographic regions. This species is known to be distributed *via* passive migration of individuals, e.g., by dispersal *via* water birds [20, 21]. The haplotype patterns of *G. lacustris* specimens observed may mirror recent random dispersal of the species, e.g. by birds from southern water bodies to the north. Additionally, low heterogeneity of sequences in each of the studied water bodies and the clearly distinct haplotype patterns in each sampling site (Fig. 5) indicate infrequent exchange of *G. lacustris* between the studied sites.

Cryptic species diversity may typically occur in ancient lakes with high degrees of endemicity as this was also found e.g. for amphipods from another ancient lake, Lake Ohrid in North Macedonia and Albania [22]. A high degree of intraspecific genetic differentiation within one species, on the other hand, was found on a small geographical scale for the amphipod species *Gammarus fossarum* that is widely distributed [23].

### Are *E. verrucosus* and *E. vittatus* species complexes comprising cryptic (sub)species?

*Eulimnogammarus verrucosus* specimens from the different regional haplotypes showed some differences in morphology, such as a different amount of setae on the dorsal part of the metasome and the urosome, and in colouring, such as continuous or intermittent black stripes along the caudal edges of the body segments (see above). However, overall the specimens from the different haplogroups were morphologically similar and based on their characteristics could morphologically clearly be assigned to this species. Furthermore, the habitats where the specimens in the different regions were sampled were corresponding and it can be assumed that their respective ecological roles are equal. Yet, the genetic differences between specimens from the different regions were as large as between species and ABGD analyses confirmed that the degree of separation of the different haplogroups was indeed on the species level.

For amphipod species of the genus *Gammarus* a threshold of 3% genetic divergence of COI sequences was suggested to be applied as indication for separate species [24]; thus 13 and 10% genetic divergence in *E. verrucosus* and *E. vittatus*, respectively, would indicate a species status of the different haplogroups. On the other hand, when the occurrence of mating of individuals was used as criterion for distinguishing between amphipod species, a “species” comprised individuals with even greater genetic divergences: mating of amphipod individuals from different molecular operational taxonomic units took place when genetic divergences were up to 21.5% [25] and 16% [26]. Thus, whether the location-specific *E. verrucosus* and *E. vittatus* haplogroups are distinct species can at this point not be determined. However, our data indicate that the species “*E. verrucosus*” and “*E. vittatus*” comprise at least three and two, respectively, cryptic (sub) species each and the species names represent complexes of similar, separated (sub) species that kept corresponding ecological and similar morphological characteristics. Further analyses of molecular data, such as multi-locus/whole-genome sequencing, and/or experimental evidence for the capability of reproduction of individuals across haplotypes would indicate whether individuals from the different haplogroups indeed belong to different species.

The separation into geographical subgroups within *E. verrucosus* as indicated by 18S rDNA sequence comparison, which was concordant with the results of the COI analyses, can be seen as further indication of ongoing speciation [27]. The mutation rate of mitochondrial DNA is known to be about one order of magnitude above that of nuclear DNA [28]; maximum sequence divergences of 13% for COI and of 0.9% for 18S rDNA within *E. verrucosus* indicate according differences in mutation rates in the examined amphipod species.

A high level of intraspecific divergences was also indicated by differences of COI sequences found for deep-water amphipod species from Baikal [5]. In a recent study exploring the phylogeny of the Baikalian amphipod fauna based on partial transcriptomes of 64 species high intraspecific diversity was indicated by sequence differences between individuals of certain species from different sampling points [7]. A high level of cryptic speciation in Lake Baikal amphipods was hypothesized by Väinölä and Kamaltynov (1999) [29] which in line with the previous results is also indicated by our data.

### Rivers as dispersal barriers in Baikal

The evidence obtained here for a clear separation of geographically defined species subgroups based on specimens from a relatively large number of sampling points indicates geographical structures/features forming insurmountable dispersal barriers for *E. verrucosus* and *E. vittatus*.

Geographical structures separating *E. verrucosus* and *E. vittatus* haplotypes are the outflow of the Angara River in the southwest and, presumably, the Angarskiy Sor in the north and the Selenga River delta in the southeast (refer to maps in Fig. 1; Additional file 4: Figure S1). Furthermore, the Sarma River delta may to some degree separate *E. verrucosus* haplotypes from the shoreline in the region between the Onguryon settlement and Severobaikalsk on the northern part of the western shore from the other haplotypes from the western shore (Fig. 5).

At its outflow, the Angara River is about 1 km wide (distance between the sampling points Lystvyanka and Port Baikal) with a water flow of at least 1–2 m/s and a maximum water depth of about 5 m which may preclude the crossing of the Angara River outflow by the amphipods. The Angarskiy Sor and the Selenga River delta are at their transitions into Baikal approximately 20 km and 50 km, respectively, wide. Their waters differ in characteristics to the water of Baikal; they are eutrophic to a much higher extent [30–32] and the sandy areas where the waters of the inflowing rivers and of Baikal intermix may be unsuitable habitats for the littoral Baikal amphipod species.

### Species-specific aspects of the intraspecific genetic diversity of the *Eulimnogammarus* species

Our finding of distinct haplotype clusters and considerable genetic diversity within *E. verrucosus* and *E. vittatus* in contrast to *E. cyaneus* was unexpected. *Eulimnogammrus cyaneus* is phylogenetically more ancient than *E. verrucosus* and *E. vittatus* [6, 7] and the time for *E. verrucosus* and *E. vittatus* to evolve into distinct haplotypes was shorter than for *E. cyaneus* and therefore it would seem more obvious that *E. cyaneus* shows comparatively greater intraspecific genetic diversity. Furthermore, *E. cyaneus* inhabits a comparatively narrow shore strip with relatively shallow water close to the shoreline [33], so that it may be expected that also smaller geographical structures, such e.g. boulders, at the shoreline act as dispersal barriers confining distinct populations of this species but not of *E. verrucosus* and *E. vittatus*. However beyond dispersal/migration important for gene flow between populations the observed differences in the genetic structures between the *Eulimnogammarus* species may also be due to factors related to mutation, selection and genetic drift [34].

The comparison of coalescent models assuming strict or relaxed molecular clocks shows no difference in mutation rate within the species pairs *E. verrucosus* / *E. cyaneus* and *E. vittatus* / *E. cyaneus*. Values of dN/dS show that the analyzed COI fragment is under equal purifying selection in the three *Eulimnogammarus* species. The comparison of ENC values indicates that *E. verrucosus* and *E. cyaneus* have similar effective population sizes, so genetic drift within populations of these species should also be similar. This indicates that the relative COI homogeneity of *E. cyaneus* compared to *E. verrucosus* and *E. vittatus* is due to species-specific adaptations to current or very recent conditions of Baikal, e.g. shoreline structures related to different water levels of the lake [35], enabling undisrupted gene flow of *E. cyaneus* across the lake.

Dispersal and therefore gene flow of Baikal amphipod species may be related to the species-specific range within environmental conditions, such as temperature, oxygen levels, water chemistry etc. that can be tolerated. Thus, *E. cyaneus* is considerably more tolerant to adverse environmental conditions than *E. verrucosus* and *E. vittatus* that are adapted to a comparatively narrow range of environmental conditions [36–39] and the narrower window of environmental conditions, such as e.g. temperature, tolerated by *E. verrucosus* and *E. vittatus* may preclude their dispersal along an environmental condition gradient that will not act as dispersal barrier for *E. cyaneus*.

Differences in dispersal of the studied *Eulimnogammarus* species across Baikal may be related to species-specific migration habits. Thus, the habitat of *E. cyaneus* is a strip of shallow water along the water edge forcing the species to migrate horizontally resulting in higher dispersal and connectivity of *E. cyaneus* populations, whereas *E. verrucosus* and *E. vittatus* inhabiting a wider range of water depths may tend to migrate vertically rather than horizontally [39]. Furthermore, as it inhabits shallow water *E. cyaneus* is in close contact to the ice cover in winter, which may act as a corridor for migration along the shoreline.

Dispersal of *E. cyaneus* *via* water birds may also be assumed but seems unlikely as population genetics data for *G. lacustris* suggest that such passive migration of amphipods is rare in the region and may not result in the homogenous haplotype distribution as found for *E. cyaneus*.

### Time of separation of *E. verrucosus* haplogroups

The observed intraspecific differences of the COI sequence can be used for an estimate for the time of separation of the different haplogroups. Commonly, the estimate for the COI divergence rate per 1 million years is 2–3%, which for deep-water Baikal amphipods was assumed to be roughly five-fold higher [5] and a generally five-fold elevated rate of speciation of Baikal amphipods in comparison with the non-Baikal amphipod taxon *Gammaridae* was suggested by Naumenko et al. (2017) [7]. When thus roughly assuming a COI mutation rate of 10–15% per 1 million years the different genetic groups within *E. verrucosus* were separated for ~1 million years.

## Conclusions

Our data provide the first clear evidence for formation of cryptic (sub)species within endemic littoral amphipod species of Lake Baikal and point to the inflows/outflow of large rivers as dispersal barriers. Under the precondition that littoral species were distributed across the entire lake interruptions of the littoral habitat, such as river inflows/outflows, are sufficient for *E. verrucosus* and *E. vittatus* that are narrowly adapted to certain environmental conditions to form genetically separate populations and eventually separate species. On the contrary, the also littoral *E. cyaneus* is or was recently able to maintain gene flow across the lake enabled by species-specific behavioral and/or physiological adaptations.

## Materials and methods

### Investigated species

The investigated species (Fig. 2) *Eulimnogammarus verrucosus* (Gerstfeldt, 1858), *E.* (subgenus *Philolimnogammarus* Bazikalova) *cyaneus* (Dybowsky, 1874) and *E.* (subgenera *Philolimnogammarus* Bazikalova) *vittatus* (Dybowsky, 1874) occur across Lake Baikal and in the Angara River, the only outflow of Baikal. Their habitat is substrate consisting of pebble stones in the shore region to water depths of mainly down to 1 m, 12 m and 30 m for *E. cyaneus*, *E. verrucosus* and *E. vittatus*, respectively [15, 33, 40]. *Gammarus lacustris* Sars, 1863 (Fig. 2) is distributed across the Holarctic; it is found in waters adjacent and even connected to Baikal but not in shore regions directly facing the open Baikal. All species are omnivorous and part of the benthic communities in the littoral zones. *Eulimnogammarus verrucosus* and *E. vittatus* belong to the winter-reproducing complex; reproduction of *E. cyaneus*, as of *G. lacustris*, takes place in summer. Morphology and coloring of the species are very characteristic and distinctive enabling unequivocal species determination of individuals based on body features [11, 15] (refer to Fig. 2).

*Eulimnogammarus verrucosus* is a large amphipod (body length of adults: 30–40 mm), the coloring is characteristic with black (along the caudal edge of each body segment) and intermittent yellow or green stripes. The period of reproduction begins in October–January and lasts until May when water temperatures are close to 0 °C [41, 42]. In the summer months only juveniles inhabit the shallow water close to shore, which can show large daily temperature fluctuations; the thermosensitive adults with an experimental thermal preference of 5–6 °C [36] generally migrate to deeper, cooler waters [33, 39].

*Eulimnogammarus vittatus* adults have body lengths of 18–20 mm with brown and orange coloration. Reproduction was found to take place from November until June [42]. The species is thermosensitive with preferred water temperatures of 5–6 °C [36]. As for *E. verrucosus*, *E. vittatus* adults migrate to deeper, cooler waters in the summer [33].

*Eulimnogammarus cyaneus* adults have body lengths of 11–15 mm. The species is characterized by a uniform blue coloring of the body surface. It is reproductively active from May to September [42]. This thermotolerant species inhabits the upper littoral zone with temperature fluctuations of up to 10 °C with maximum temperatures of up to 25 °C [37]; the preferred temperature range is around 11–12 °C [36].

*Gammarus lacustris* (body length of adults: 20–25 mm) is widespread in a variety of lentic ecosystems with different environmental conditions across the Holarctic. In the Baikal region the species is found in sheltered bays (“sors”) of the lake and in shallow ponds around the lake. The reproduction period is in the summer [11]. The preferred temperature of this thermotolerant species is 15–16 °C [36].

Species determination of the collected specimens was performed based on the keys in [11, 15]. The sampled species are neither endangered nor protected, and specific permissions for samplings were not required.

### Animal samplings and sampling sites

*Eulimnogammarus verrucosus*, *E. vittatus* and *E. cyaneus* specimens were from various sampling campaigns in 2011–2018 and were obtained by kick-sampling in the littoral zone of Lake Baikal at water depths of 0–1 m at overall 22 locations along the shorelines around the lake, of Olkhon Island (western shore towards Maloe More Strait and eastern shore towards open Baikal) and of Bolshoy Ushkaniy Island (Fig. 1; Additional file 4: Figure S1; Table 1; Additional file 1: Table S1). Distances between sampling sites were from < 1 km to up to 600 km; the sampling locations on the Pokoiniki Cap (see Additional file 1: Table S1), as well as at Bolshie Koty Bay and Warnachka Bay were separated only by several hundred meters. *Eulimnogammarus verrucosus* and *E. cyaneus* specimens were obtained from 21 and 17 sampling locations, respectively. Specimens from these two species were sampled repeatedly in different years at the sampling locations in Bolshie Koty Bay and Maloe More Strait for investigating the genetic diversity of the respective populations over time (see Additional file 1: Table S1). Overall, 216 *E. verrucosus* and 155 *E. cyaneus* specimens were investigated (Table 1). *Eulimnogammarus vittatus* individuals were obtained in 2017 from the sampling points at Bolshie Koty Bay, at Listvyanka Bay and at Baikalsk city. A total of 22 individuals were examined (Table 1). *Gammarus lacustris* was collected from a small and shallow artificial lake close to the Bolshie Koty settlement (“Lake No 14”); a location in the delta formed by the Upper Angara and Kichera Rivers (Angarskiy Sor) close to the city of Nizhneangarsk; and a water body close to the Onguryon settlement (Fig. 1; Additional file 1: Table S1). The number of investigated *G. lacustris* individuals was 32. The Onguryon sampling points for *G. lacustris* and *E. verrucosus* / *E. cyaneus* are not identical: the small pond where *G. lacustris* was sampled is only within a few meters from Baikal but not inhabited by Baikal amphipods; the Onguryon sampling point at Baikal is inhabited by Baikal species, such as *E. verrucosus* and *E. cyaneus*, but not by *G. lacustris*.

Immediately after sampling, the amphipods were placed in 70% ethanol for several minutes, transferred to 99% ethanol and stored in liquid nitrogen. Before long-term storage of samples at − 80 °C, the ethanol was exchanged.

### DNA extraction, PCR and sequencing

DNA was extracted from ~ 50 mg amphipod tissue (extremities of the larger species *E. verrucosus* and *E. vittatus*, about 2/3 of the whole body from *E. cyaneus* and *G. lacustris*) using the DNeasy Blood & Tissue Kit (Qiagen, Germany) according to the manufacturer’s instructions.

Polymerase chain reactions (PCR) were performed with GoTaq Flexi DNA Polymerase (Promega, USA). Primers were universal COI primers from [43] (F: HCO2198; R: LCO1490) and primers designed against COI sequences in the mitochondrial genomes of *E. verrucosus* [44] (F: Eve_F1, Eve_F3; R: Eve_R1, Eve_R2, Eve_R3) and of *G. duebeni* (F: Gdu_F2; JN704067.1; codes here and below are sequence accession numbers from the National Center for Biotechnology Information, NCBI):

HCO2198: TAAACTTCAGGGTGACCAAAAAATCA.

LCO1490: GGTCAACAAATCATAAAGATATTGG.

Eve_F1: TCTCTACTAATCATAAAGATATCGG.

Gdu_F2: TCTCAACAAACCATAAAGACATCGG.

Eve_F3: AGAATAATCGGTACCTCTATAAGG.

Eve_R1: TAAACTTCTGGATGGCCAAAGAATCA.

Eve_R2: GATTCTTGTCTTACGATATGAGAG.

Eve_R3: GATTATGCCGAATGCAGGGAGGATG.

Primers were used in the following combinations:

*E. cyaneus*: HCO2198/LCO1490.

*E. verrucosus*: HCO2198/LCO1490; Eve_F1/Eve_R1; Gdu_F2/Eve_R2; Eve_F3/Eve_R3.

*E. vittatus*: HCO2198/LCO1490; Eve_F1/Eve_R1.

*G. lacustris*: Eve_F1/Eve_R2.

The *E. verrucosus* 18S rDNA fragment was amplified with the following primers [16]:

18SF: CCTACTGGTTGATCCTGCCAGT.

18S700R: CGCGGCTGCTGGCACCAGAC.

The PCR products were purified with the GeneJET PCR Purification kit (Thermo Fisher Scientific, USA) and sequenced in both directions with a Genetic Analyzer 3130xl sequencer (Applied Biosystems, USA) using standard cycle sequencing protocols (BigDye® Terminator v3.1 Cycle Sequencing kit; Life Technologies, USA) with the respective COI/18S rDNA PCR primers.

### Analysis of DNA sequences

Sequence reads were assembled with DNADynamo (BlueTractorSoftware, UK) to obtain COI consensus sequences for each individual. The COI sequences were excluded if only one read was obtained per individual or if the quality of the obtained sequences was too low and the obtained length of a sequence for an individual was below the length of the COI sequence fragment that was used for the analysis. For 18S rDNA also single reads were included in the analysis. Sequences were trimmed to remove primer sequences and to obtain uniform sequence lengths; COI sequence lengths were 603 bp for *E. verrucosus*, 631 bp for *E. cyaneus*, 640 bp for *E. vittatus* and 662 bp for *G. lacustris*. Available COI sequences of the endemic Baikal amphipod *Gmelinoides fasciatus* (Stebbing, 1899) were included in the COI sequence analysis (FJ715823.1 - FJ715903.1, FJ715905.1, FJ715919.1) [9]. Homology of the COI sequences was confirmed by aligning them to the *E. verrucosus* COI gene sequence (KF690638.1, 1634–3167); the obtained fragments corresponded to the following positions in the *E. verrucosus* COI gene sequence: 97–699 for *E. verrucosus*, 60–699 for *E. vittatus*, 69–699 for *E. cyaneus*, 94–755 for *G. lacustris* and 107–671 for *G. fasciatus*. The *E. verrucosus* 18S rDNA was trimmed to a length of 472 bp. Sequences were aligned with ClustalW [45] in UGENE (UniPro, Russia) [46].

The percentage differences in nucleotides (Hamming dissimilarity, uncorrected) in COI sequence fragments as a measure for genetic distances of specimens were calculated in UGENE excluding the gaps if necessary. Possible species separation processes were determined with ABGD (“Automatic Barcode Gap Discovery”) [47] with default settings using the COI data of the four species. dN/dS ratios were calculated for the obtained COI sequences and sequences of *G. fasciatus* in MEGA7 version 7.0.26 [48] using HyPhy [49] with the HKY85 model (determined using the internal MEGA tool). dN and dS values were summarized over all complete codons of the COI fragments to obtain the dN/dS ratio for each species.

The effective number of codons (ENC), a metric of codon usage bias, was calculated with CodonW (version 1.4.4; application of the “Insects and Plathyhelminthes mitochondrial code”) independently for *E. verrucosus* and *E. cyaneus* specimens from the western (region between Listvyanka Bay and the city of Severobaikalsk), southern (region between Port Baikal and the Kluevka settlement) and eastern (region between the Ust-Barguzin settlement and Davsha Bay) sampling location areas to assess relative effective population sizes of these species as suggested previously [12]. Statistical significance of differences in ENC within each species was assessed by pairwise comparisons using the Mann-Whitney U test with the Hommel correction in R [50]. Interspecies comparisons of ENC values of amphipod specimens from the sampling location areas were performed identically.

Spatial analysis of molecular variance implemented in SAMOVA 2.0 [51] was applied to identify groupings of *E. verrucosus* sampling points along the west coast (from Listvyanka to Severobaikalsk) and of *E. cyaneus* sampling points across the entire Lake Baikal into genetically differentiated populations. The analysis was run with 100 independent simulated annealing processes and the number of geographic groupings from *K* = 2 to 8 for *E. verrucosus* and 2 to 15 for *E. cyaneus*; other options were set to the default values. The maximum *F*_CT_ value resulting from SAMOVA with different *K* values generally indicates the optimal number of groups *K* [51].

The phylogenetic networks were built using SplitsTree4 version 4.14.6 [52] with default parameters (UncorrectedP, NeighborNet, EqualAngle) and displayed using the phangorn package [53] in R [50]. To produce the COI phylogenetic network including all studied species, the alignment was trimmed to match the shortest sequence fragment which was that of *E. verrucosus*. The corresponding geographical maps (Figs. 1, 4, 5, 6) were prepared using the R package sf and cartographic data provided by the Natural Earth project (http://www.naturalearthdata.com/). The cartographic data from Natural Earth are freely available and may be used for all personal, educational, and commercial purposes (https://www.naturalearthdata.com/about/terms-of-use/).

The maximum likelihood trees were built with IQ-TREE version 1.6.10 [54] with the substitution model auto-fitted with ModelFinder [55]; Shimodaira-Hasegawa approximate likelihood ratio test (aLRT) with 1000 bootstrap replicates and a Bayesian-like transformation of aLRT [56]. The trees were visualized with ggtree version 1.14.6 [57].

### Pair-wise comparison of mutation rates

Species-specific relative mutation rates within the studied COI fragment were compared within the pairs *E. cyaneus / E. verrucosus* and *E. cyaneus / E. vittatus*; the COI sequence of the Baikal endemic amphipod *Brachyuropus grewingkii* served as outgroup (NC_026309.1) [6] (similar to the approach of Tajima’s relative rate test). For this analysis, the COI sequences were selected that represented the variability in each cluster of haplotypes found in the phylogenetic networks with up to ten  sequences per each main haplogroup of all three *Eulimnogammarus* species.

Mutation rates were analyzed with BEAST version 1.8.4 [58] sequentially using the options “Strict clock” and “Fixed local clock” (relaxed molecular clock model allowing different substitution rates in different taxa) using identical settings. Sequences of each *Eulimnogammarus* species were assigned to the corresponding taxon and all sequences of either *E. verrucosus* and *E. cyaneus* or *E. vittatus* and *E. cyaneus* together were additionally assigned to the same taxon. The substitution model suggested by jModelTest [59] was HKY85 set with empirical base frequencies and the gamma site heterogeneity model with four gamma categories. The “Coalescent: Bayesian Skyline” model was used as tree prior with ten groups and piecewise-linear Skyline model. The distribution of mutation rates was set to log-normal, and the length of chain was 10,000,000. The obtained effective sample sizes for parameters “likelihood” and “treeLikelihood” visualized in Tracer 1.7.0 [60] were always higher than 1000, and effective sample sizes of all mutation rates were over 180.

The plausibilities of the obtained models assuming strict or relaxed molecular clock were compared using marginal likelihood estimation by path sampling and stepping-stone sampling methods also in BEAST. The number of path steps was set to 50, and the length of the chain was 5,000,000. The effective sample size of “pathLikelihood.delta” was always over 100 with these parameters. The Bayes factor between the probabilities of relaxed clock and strict clock models (BF_RS_) was calculated as the *e* in power of difference between obtained log marginal likelihoods.

### Morphological examinations

Detailed morphological examinations of *E. verrucosus* individuals from different sampling sites were performed in 2018 with intact specimens stored in ethanol upon samplings in 2012, 2014 and 2018. Specimens were examined for morphological features based on [15] under a stereomicroscope (LOMO, Russia).

## Additional files

Additional file 1: **Table S1.** Information on sampling sites with names, coordinates, water parameters, years of samplings and numbers of COI/18S rDNA sequences per species. (DOCX 21 kb)

Additional file 2: **List of COI sequences S2.** Alignment of COI sequences from all specimens from all examined amphipod species. From this study are sequences from *E. verrucosus*, *E. vittatus*, *E. cyaneus*, *G. lacustris*. From NCBI are sequences from *E. maackii* (AY926663.1), *E. viridulus* (AY926665.1), *E. viridis* (AY926664.1), *Brachyuropus grewingkii* (NC_026309.1), *G. lacustris* (AY926671.1; JF965916.1; GU066811.1). (TXT 308 kb)

Additional file 3: **List of 18S rDNA sequences S3.** Alignment of 18S rDNA sequences from *E. verrucosus* specimens from this study and from NCBI (AY926773.1). (TXT 7 kb)

Additional file 4: **Figure S1.** Bathymetric map of Lake Baikal. The 3D map shows a detail with the Akademicheskiy Ridge separating the northern and southern basins of Baikal. The water depths are indicated by different shades of blue. Note that regions both slightly below and slightly above the current water level are indicated in light blue on the 3D map. The maps are freely available at http://dataservices.gfz-potsdam.de/SDDB/showshort.php?id=escidoc:76692 and [64] and may be used and publicly distributed. (PNG 1195 kb)

Additional file 5: **Figure S2.** Maximum likelihood tree based on the alignment of the corresponding COI sequence fragments from the four studied species *Eulimnogammarus verrucosus*, *E. vittatus*, *E. cyaneus* and *Gammarus lacustris*. Complementary to Fig. 3. The numbers near the nodes signify SH-aLRT bootstrap values and approximate Bayes posterior probabilities, respectively. (PDF 43 kb)

Additional file 6: **Figure S3.** Maximum likelihood tree based on 18S rDNA sequence fragment from *E. verrucosus* specimens from sampling points in the southern, western and eastern regions of Baikal. Complementary to Fig. 6. 18S rDNA sequences of *B. grewingkii* (FJ752386.1) and *E. viridis* (AY926774.1) were used as outgroups. The numbers near the nodes signify SH-aLRT bootstrap values and approximate Bayes posterior probabilities, respectively. (PDF 5 kb)

## Abbreviations

ABGD: Automatic Barcode Gap Discovery
aLRT: approximate Likelihood Ratio Test
BF: Bayes Factor
bp: base pairs
COI: Cytochrome c Oxidase I
ENC: Effective Number of Codons
rDNA: ribosomal DNA
