# Supplementary material for: Indication of ongoing amphipod speciation in Lake Baikal by genetic structures within endemic species
Source: BMC Evol Biol. 2019 Jul 8;19:138. doi: 10.1186/s12862-019-1470-8 (PMC6613252; doi:10.1186/s12862-019-1470-8)
Supplement: Supplementary file 1 — Table S1. Information on sampling sites with names, coordinates, water parameters, years of samplings and numbers of COI/18S rDNA sequences per species. (DOCX 21 kb) [file 12862_2019_1470_MOESM1_ESM.docx]

Table S1. Information on sampling sites with names, coordinates, water parameters, years of samplings and numbers of COI/18s rRDNA sequences per species. Year of sampling of *E. vittatus* is always 2017. Abbreviations: cons — consensus sequence, forw — forward sequence, rev — reverse sequence.

| № | Location | Coordinates, N | Coordinates, E | Water parameters: T (°C); [O_2_] (mg/l); pH; EC (µS/cm) | Year of sampling | Number of COI sequences – *E. verrucosus* | Number of COI sequences – *E. vittatus* | Number of COI sequences – *E. cyaneus* | Number of COI sequences – *G. lacustris* | Number of 18S rDNA sequences – *E. verrucosus* |
| --- | --- | --- | --- | --- | --- | --- | --- | --- | --- | --- |
| 1 | Baikalsk | 51°31'43.75" | 104°9'3.30" | 16; 10.65; 7.8; 117 | 2012 | 11 | 7 | 8 |  | 1 cons + 1 rev |
| 2 | Baikalskoe | 55° 21'25" | 109°11'57.8" | 8 | 2014 | 11 |  | 10 |  |  |
| 3 | Bolshie Koty | 51°54'11.67" | 105°4'7.61" | 11; 12; 7.5; 117 | 2011-2013 | 32 | 9 | 19 |  |  |
| 4 | Bolshoy Ushkaniy Island | 53° 51'1.61" | 108°39'27.6" | 7 | 2014 | 11 |  | 10 |  |  |
| 5 | Davsha | 54°21'21.63" | 109°29'55.74" |  | 2016 | 6 |  |  |  |  |
| 6 | Kluevka | 51°41'39.02" | 105°45'43.20" |  | 2014 |  |  | 8 |  |  |
| 7 | Kotelnikovskyi | 55°04'0.12" | 109°06'24.2" | Range from 20 °C and lower | 2014 | 10 |  | 3 |  |  |
| 8 | Kultuk | 51°43'45.02" | 103°43'42.69" | 10; 10.3, 6.8; 121 | 2012 | 12 |  |  |  | 2 cons |
| 9 | Lake 14 | 51°55'14.39" | 105°4'19.48" | 8; 9; 6.8 | 2012 |  |  |  | 11 |  |
| 10 | Listvyanka | 51°52'14.07" | 104°49'41.78" | 6.6; 12.8; 8.3; 119 | 2012 | 11 | 6 | 6 |  | 2 cons |
| 11 | Maloe More (west of Olkhon Island) | 53°12'12.88" | 107°20'37.80" | 10; 15.3; 7.38; 117 | 2012, 2013 | 21 |  | 19 |  |  |
| 12 | Nizhneangarsk | 55°50'44.42" | 109°40'6.90" | 22; 10; 10.5; 69 | 2012 |  |  |  | 11 |  |
| 13 | Olkhon Island (east) | 53°4'2.75" | 107°18'52.50" | 8; 11.8; 7.7; 119 | 2012 | 8 |  |  |  |  |
| 14 | Onguryon | 53°37'01.0" | 107°37'00.4" | 10 °C for Eve and Ecy and 17 °C for Gla | 2014 | 10 |  | 11 | 10 |  |
| 15 | Pokoiniki, "Solnechnaya" | 54°01'47.8" | 108°15'24.6" | 7 | 2014 | 5 |  | 8 |  |  |
| 16 | Pokoiniki, shallow bay | 54°0'42.51" | 108°14'35.39" | 19 | 2014 | 1 |  | 2 |  |  |
| 17 | Port Baikal | 51°52'14.65" | 104°48'42.47" | 5.6; 11.52; 7.92; 120 | 2012 | 10 |  | 11 |  | 1 cons |
| 18 | Severobaikalsk | 55°37'5.24" | 109°21'14.45" | 16.5; 11.4; 7.5; 113 (21.07.2012)  17.6; 10.75; 6.93; 106 (24.07.2012) | 2012 | 13 |  | 10 |  | 2 cons |
| 19 | Solontsovyi | 54°07'0.32" | 108°17'4.23" | 7 | 2014 | 11 |  | 2 |  |  |
| 20 | Svyatoy Nos | 53°41'29.60" | 108°41'46.37" | 13 | 2014 | 11 |  | 10 |  | 2 cons |
| 21 | Ulan-Nur | 52°50'00" | 106°42'33.2" | 7 | 2014 | 3 |  | 8 |  |  |
| 22 | Ust-Barguzin | 53°22'42.26" | 108°58'55.20" |  | 2016 | 6 |  |  |  |  |
| 23 | Warnachka | 51°54'13.10" | 105°6'6.96" |  | 2013 | 9 |  |  |  | 1 cons + 1 forw |
| 24 | Zavorotnaya | 54°17'1.91" | 108°28'3.75" | 7 | 2014 | 5 |  | 10 |  |  |
