# Supplementary figures and images for: Indication of ongoing amphipod speciation in Lake Baikal by genetic structures within endemic species
Source: BMC Evol Biol. 2019 Jul 8;19:138. doi: 10.1186/s12862-019-1470-8 (PMC6613252; doi:10.1186/s12862-019-1470-8)

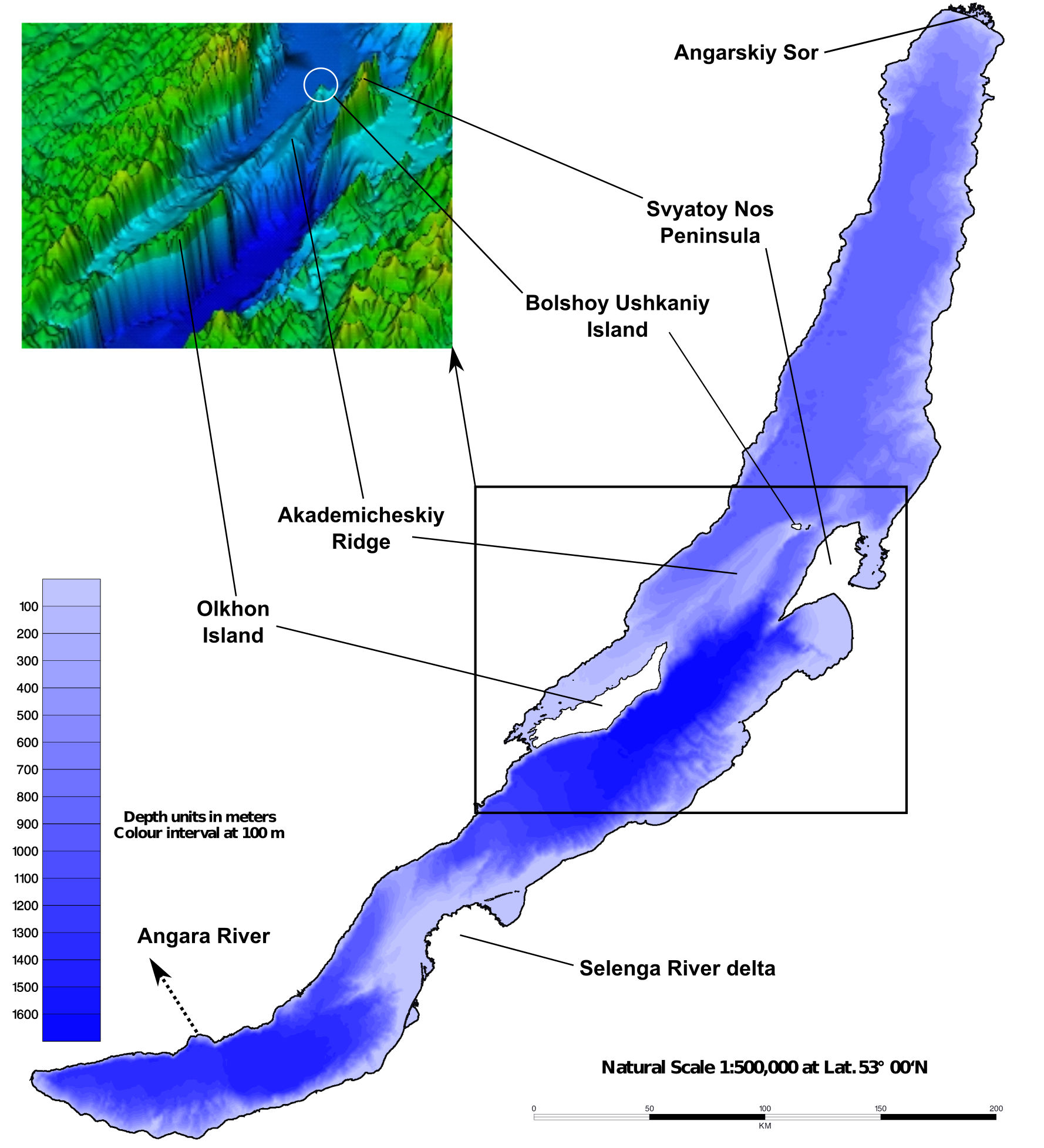

Supplement: Supplementary file 4 — Figure S1. Bathymetric map of Lake Baikal. The 3D map shows a detail with the Akademicheskiy Ridge separating the northern and southern basins of Baikal. The water depths are indicated by different shades of blue. Note that regions both slightly below and slightly above the current water level are indicated in light blue on the 3D map. The maps are freely available at http://dataservices.gfz-potsdam.de/SDDB/showshort.php?id=escidoc:76692 and [64] and may be used and publicly distributed. (PNG 1195 kb) [file 12862_2019_1470_MOESM4_ESM.png]

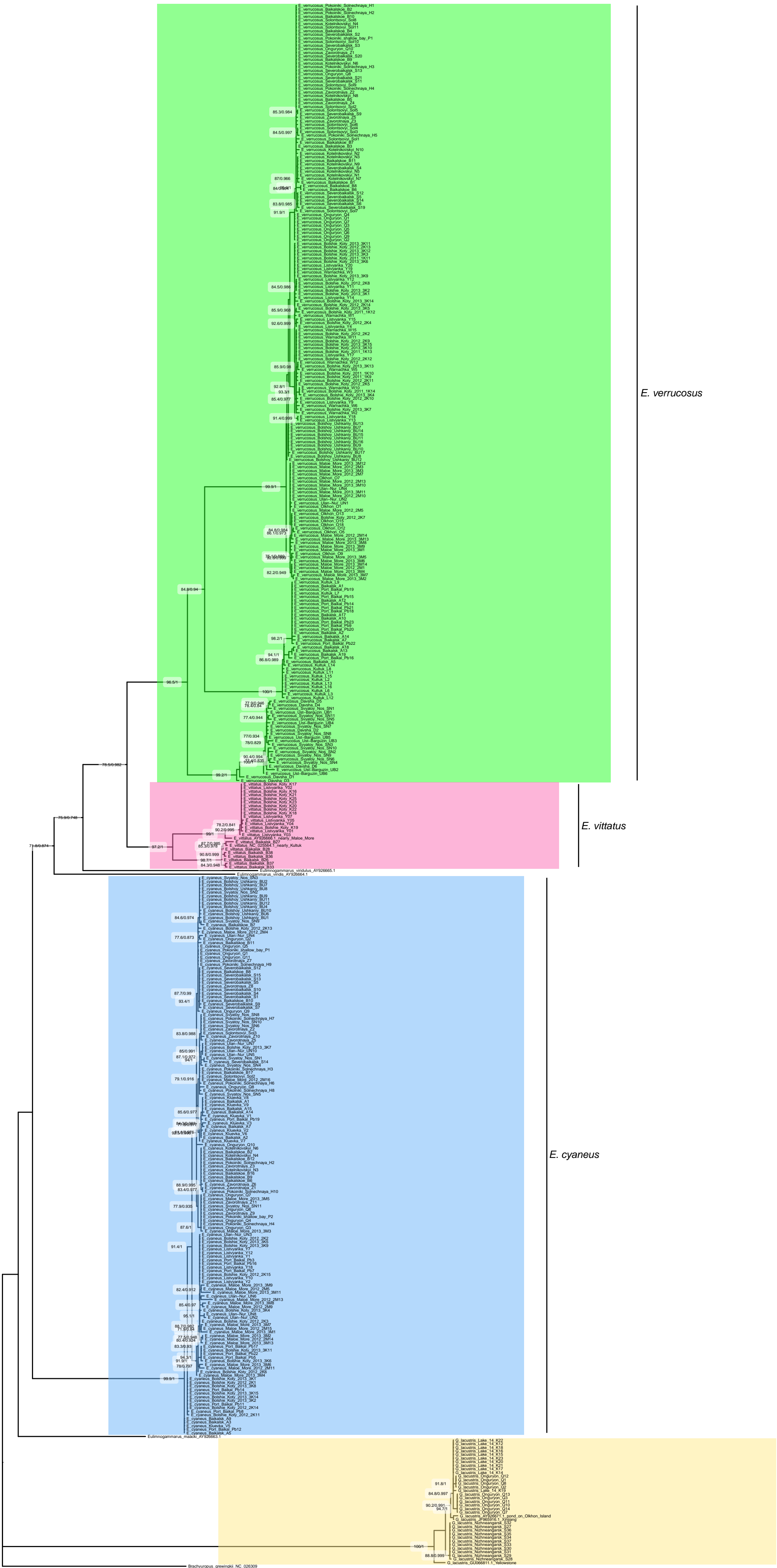

*E. verrucosus*

*E. vittatus*

*E. cyaneus*

*G. lacustris*

Supplement: Supplementary file 5 — Figure S2. Maximum likelihood tree based on the alignment of the corresponding COI sequence fragments from the four studied species Eulimnogammarus verrucosus, E. vittatus, E. cyaneus and Gammarus lacustris. Complementary to Fig. 3. The numbers near the nodes signify SH-aLRT bootstrap values and approximate Bayes posterior probabilities, respectively. (PDF 43 kb) [file 12862_2019_1470_MOESM5_ESM.pdf]

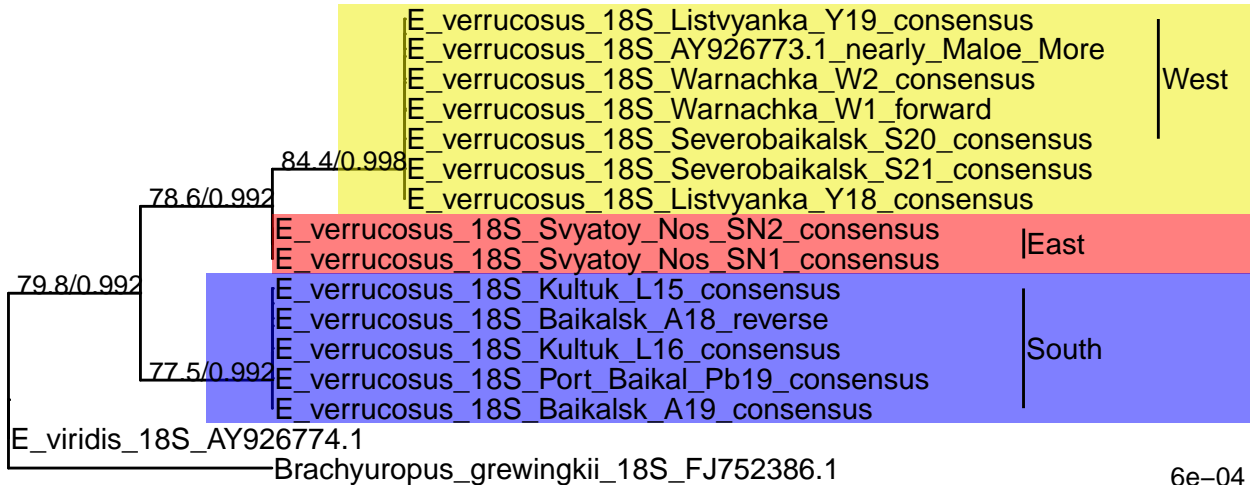

Supplement: Supplementary file 6 — Figure S3. Maximum likelihood tree based on 18S rDNA sequence fragment from E. verrucosus specimens from sampling points in the southern, western and eastern regions of Baikal. Complementary to Fig. 6. 18S rDNA sequences of B. grewingkii (FJ752386.1) and E. viridis (AY926774.1) were used as outgroups. The numbers near the nodes signify SH-aLRT bootstrap values and approximate Bayes posterior probabilities, respectively. (PDF 5 kb) [file 12862_2019_1470_MOESM6_ESM.pdf]
